# Endocarditis: Rising Incidence in the Post-COVID-19 Pandemic Era: A Narrative Review

**DOI:** 10.3390/jcm14207274

**Published:** 2025-10-15

**Authors:** Cynthia L. Lefter, Salvatore Poddi, Alessio Rungatscher

**Affiliations:** 1 George Emil Palade University of Medicine, Pharmacy, Science and Technology, 540139 Targu Mures, Romania; cynthia_lefter@yahoo.com; 2 Division of Cardiac Surgery, University of Verona Medical Center, 37126 Verona, Italy; salvatore.poddi@univr.it

**Keywords:** infective endocarditis, non-bacterial thrombotic endocarditis, COVID-19, pandemic, incidence, echocardiography, diagnosis

## Abstract

Infective Endocarditis (IE) incidence has increased in recent years, driven by emerging risk factors affecting both elderly and young adults. Also, the role of Non-Bacterial Thrombotic Endocarditis (NBTE) is gaining importance as it is a subtle, probably underdiagnosed entity. Moreover, the COVID-19 pandemic has influenced the epidemiology of endocarditis, raising questions about their relationship, diagnosis, and management. Diagnosis of IE is sometimes challenging, and classic criteria are now being rediscussed. The aim of our study is to provide a narrative review about how and why IE incidence is rising, the role of NBTE, the impact of the COVID-19 pandemic on endocarditis patterns, and the current diagnostic challenges we face in the post-pandemic era.

## 1. Introduction

**Figure 1:**
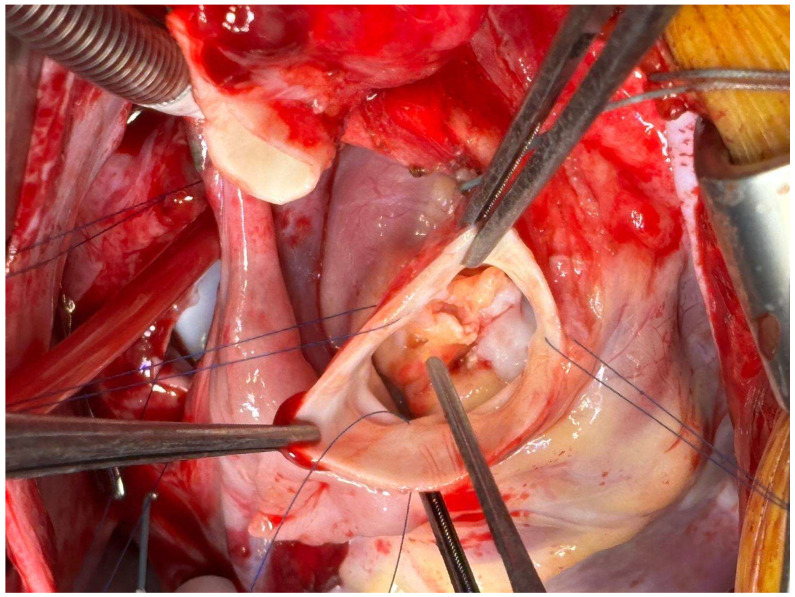
Aortic valve infective endocarditis before Aortic Valve Replacement: vegetations are visible on leaflets surface.

**Figure 2:**
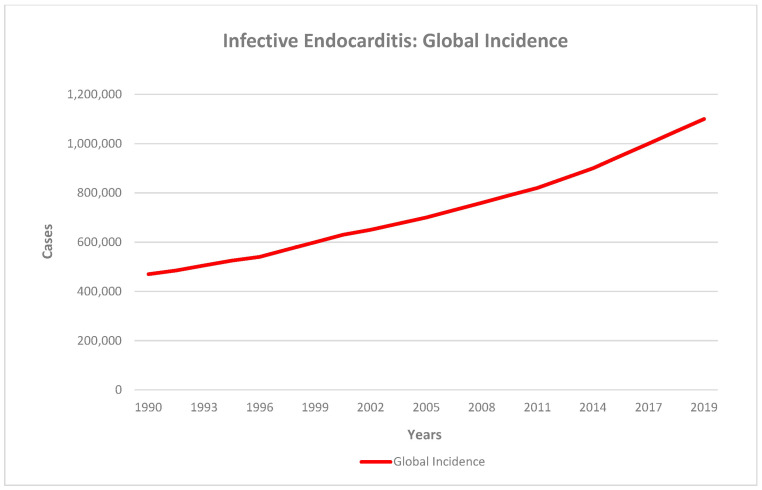
Global incidence of Infective Endocarditis (IE) 1990 through 2019. IE incidence increased globally from 478,002 cases in 1990 through 1,090,527 cases in 2019. Figure adapted from Momtazmanesh et al., 2021 [3].

Infective endocarditis (IE) is an infection of the cardiac endothelium that may affect native or prosthetic heart valves, the mural endocardium, and implanted structures such as pacemaker leads, defibrillators, or endovascular catheters (Figure 1) [1]. Although relatively uncommon, some studies estimated an incidence of 3–10 cases per 100,000 population—IE remains a severe condition that poses significant management challenges, with a 30-day mortality rate of up to 30% [2]. However, several authors have reported that its incidence has steadily increased over the past three decades. Momtazmanesh documented an increasing global incidence from 478,002 in 1990 through 1,090,527 in 2019 (Figure 2) [3]. Hammond-Haley also described a recent incidence surge in several countries (including the United States and the United Kingdom) [4].

The rising incidence of new IE cases has been driven not only by improved diagnostic capabilities (updated criteria and advanced imaging) but also by a genuine increase in risk factors: older population, increased number of patients with comorbidities or congenital heart disease (CHD) surviving into adulthood, widespread use of intracardiac or endovascular devices, central venous lines, increased exposure to invasive procedures, intravenous drug use [5,6,7].

This narrative review aims to analyze the causes and potential mechanisms underlying the increasing incidence of IE, to highlight the clinical and diagnostic particularities of Non-bacterial Thrombotic Endocarditis (NBTE), and to emphasize the essential role that the COVID-19 pandemic played in the emergence and progression of these conditions. Additionally, emerging clinical patterns and current obstacles in establishing an accurate diagnosis are discussed.

## 2. Methodology

The format of a narrative review rather than a systematic review is more suitable for this topic because of the heterogeneous nature of the current literature and the lack of high-quality studies on this emerging topic. A narrative review is a useful tool to provide a complete overview and describe a broad topic such as endocarditis. Specifically, we would like to give a viewpoint that could hopefully inspire further detailed clinical studies to address current knowledge gaps. A systematic literature search was conducted through the PubMed database to identify relevant papers published between January 2014 and September 2025. We selected the 2014–2025 interval to include recent articles and ensure an adequate pre-pandemic time frame (2014–2019). We decided to use the PubMed database as it is the most widely accessible and comprehensive source for biomedical literature, ensuring transparency and reproducibility of our approach. The following keywords and sentences were used: “infective endocarditis incidence”; “Non-bacterial Thrombotic Endocarditis”; “endocarditis NBTE”; “endocarditis after COVID-19”; “endocarditis COVID”; “NBTE COVID”; “endocarditis diagnosis”; “endocarditis challenges”. Inclusion criteria included review articles, descriptive cohort studies, case reports relevant to the topics identified through the previously listed keywords. Exclusion criteria were duplicates, non-English articles, and studies clearly outside the scope of this review. We initially found 2478 articles in total. As a first step, we read the title of each study and selected the ones that appeared relevant for our project. As a second step, we read abstracts to select the potentially interesting papers. The latter were carefully read in toto (as a third step) to select the most appropriate articles for inclusion. To avoid bias, the search and selection process was carried out by two authors independently (C.L.L. and S.P.); the process was thoroughly supervised by a third author (A.R.). The literature search and selection were performed between May 2025 and September 2025. At the end of our search and selection process, we decided to include 60 studies in our narrative review.

## 3. Incidence and Epidemiology

Hospitalization incidence for IE has been continuously increasing, as evidenced by statistical data estimating a steady increase from 11 to 15 cases per 100,000 inhabitants between 2000 and 2011 [8]. Data from the EURO-ENDO registry, which included 3116 patients from 156 centers across 40 countries, reported an increased incidence of prosthetic valve-associated cases (30.1%), Cardiac Implantable Electronic Device (CIED) infections (9.9%), and healthcare-associated infections (32.96%). A significant proportion of these infections are nosocomial (60.8%), highlighting the major role of hospital interventions as a risk factor [9].

Over the past decades, a marked increase in the mean age of IE patients has also been observed, rising from approximately 45 years in the 1980s to over 55 years in the 2000s [10]. The use and implantation of prosthetic valves and CIEDs also increased [11].

IE incidence is currently higher in men [12]. The aortic valve is the most involved among men, whereas tricuspid valve IE is more frequent in women. [13].

Patients with intravenous drug use (IDU) IE tend to be younger (mean age 38.3 years versus 50.4 years for non-IDU IE), predominantly female (45.3% vs. 41.1% male), and socioeconomically disadvantaged; they frequently present with pulmonary hypertension and liver disease, but less commonly with systemic hypertension, diabetes, renal failure [14].

An important factor to consider when interpreting IE incidence variations is the modification of antibiotic prophylaxis guidelines. Although the restriction of prophylaxis indications in the USA and France was not followed by a significant increase in IE cases, a slight rise was observed in the United Kingdom, without clear microbiological evidence to support a causal link [15].

A case-crossover study conducted in England (involving a cohort of over 14,000 hospitalized IE patients) identified significant associations between certain invasive procedures—including dental extractions (OR 2.14), CIED implantation (OR 1.54), gastrointestinal endoscopies (OR > 1.5), and bronchoscopies (OR 1.33)—and the onset of IE within three months post-procedure. Although the absolute risk remained low in the general population, it could reach nearly 50 per 100,000 dental extractions among patients with major predisposing factors [16].

The expanded use of CIEDs has significantly contributed to the rising incidence of IE. Up to 36% of patients with CIEDs and staphylococcal bacteremia develop device-related infections; similarly, left ventricular assist devices infections are frequent and associated with a 5.6-fold increase in one-year mortality [17].

Another crucial factor explaining the increased incidence of IE is the prevalence of CHD, which contributes through the complexity of anatomical defects and the numerous surgical interventions needed. A nationwide Danish study conducted over 42 years involving 23,464 CHD patients reported an IE incidence of 5.2 per 10,000 person-years, significantly higher than in the general population. This higher IE risk was associated with untreated cyanotic defects, cardiac prostheses, implantable devices, and chronic kidney disease. The most vulnerable CHD types were tetralogy of Fallot, transposition of the great arteries, univentricular heart, and valvular anomalies [18].

Another study of 36,030 CHD patients reported an IE incidence of 2% over a median follow-up of 15.5 years. Data were compared to the general population: IE risk was 54.8 times higher in CHD patients and remained 26.6 times higher after adjusting for risk factors (previous cardiac surgeries, traditional risk factors, and cardiovascular comorbidities). The highest incidence was observed in patients with complex congenital lesions (5.7%), especially those with prior IE or implantable devices [19].

## 4. Non-Bacterial Thrombotic Endocarditis (NBTE)

NBTE—also termed marantic, thrombotic, verrucous, or Libman–Sacks endocarditis in systemic lupus erythematosus (SLE)—is a rare condition characterized by sterile fibrin-platelet thrombi deposits on cardiac valves, in the absence of active infection. Often asymptomatic until embolic complications or valvular dysfunction arise, NBTE is frequently diagnosed post-mortem. The pathogenic mechanism involves endothelial injury on a procoagulant background, commonly associated with autoimmune diseases, malignancies, or hypercoagulable states such as antiphospholipid antibody syndrome (APLAS) or disseminated intravascular coagulation [20].

Despite its low prevalence, early recognition is crucial since NBTE can rapidly progress to severe complications. Diagnosis is often challenging, particularly in the context of myelosuppression or negative blood cultures, which can mask classical signs of infection. Patients may present with various embolic symptoms—ranging from hematuria, abdominal pain, or acute limb ischemia to angina, myocardial infarction, and mesenteric or splenic ischemia. Clinical manifestations are frequently nonspecific, and cardiac murmurs are rare or absent, requiring a high index of suspicion to differentiate NBTE from classic IE [21]. Inclusion in the differential diagnosis is essential in patients with comorbidities or unexplained embolic events [22].

NBTE clinical presentation is highly variable, and documented cases in the literature emphasize this heterogeneity. In one case, a 49-year-old female patient with high-grade serous ovarian carcinoma exhibited multiple systemic thromboembolic events and underwent resuscitation for ventricular fibrillation, without clinical signs or positive blood cultures for infection. Echocardiography identified non-infectious mitral vegetations, supporting a diagnosis of NBTE associated with Trousseau’s paraneoplastic thrombotic syndrome [23].

In another case, a 55-year-old man with a history of chronic tobacco and drug use presented with dyspnea, orthopnea, epigastric pain, and peripheral edema, and was diagnosed with bilateral pulmonary embolism and non-ST-elevation myocardial infarction. Abdominal imaging and liver biopsy confirmed metastatic pancreatic adenocarcinoma. Trans-esophageal echocardiogram (TEE) revealed an aortic vegetation and severe regurgitation with no signs of infection, supporting the diagnosis of NBTE [24].

Dietrich et al. described another illustrative case of NBTE mimicking early prosthetic valve infection in a young female patient with SLE and APLAS. Four months after implantation of a mitral bioprosthesis, the patient developed severe dyspnea; TEE revealed vegetations and severe stenosis. The initial suspicion was prosthetic IE, but negative cultures, sterile organized vegetations, and absence of inflammatory infiltrate on the explanted valve supported the diagnosis of NBTE [25].

One of the most comprehensive clinical studies on NBTE retrospectively analyzed 42 patients diagnosed over a 20-year period. Most patients were female (66.7%), with a mean age of 54 years, presenting significant comorbidities such as malignancies (40.5%), SLE (33.3%), and APLAS (35.7%). The most frequently associated malignant tumors were pulmonary, pancreatic, and breast adenocarcinomas. TEE demonstrated superior sensitivity (97.1%) compared to trans-thoracic echocardiography (TTE) (45.2%), with vegetations most commonly located on the mitral and aortic valves [26].

Diagnosis of NBTE is also particularly challenging when associated with Behçet’s disease (BD), where inflammatory, thrombotic, and valvular manifestations may mimic IE [27]. NBTE has also been described after COVID-19 infection [28,29].

## 5. Infective Endocarditis After COVID-19

The COVID-19 pandemic, caused by the SARS-CoV-2 virus, profoundly affected healthcare systems and cardiovascular care. Significant declines were recorded in presentations for acute cardiovascular events, delays in diagnosis and treatment, as well as increased out-of-hospital cardiovascular mortality [30]. In this context, understanding the evolution of post-COVID IE incidence is crucial, especially among younger patients with predisposing factors, suggesting a post-viral infection cardiovascular vulnerability. Some authors hypothesized a correlation between COVID-19 and IE (Table 1). Possible mechanisms include systemic and valvular endothelial inflammation that promotes bacterial adhesion (e.g., S. aureus), as well as the avoidance of investigations such as TEE during the pandemic, which may be associated with underdiagnosis of this pathology [31]. In his 2023 review, Bele concluded that COVID19-related damage (endothelial dysfunction, immune dysregulation, microbial translocation) could create a conducive environment for endocardial bacterial infection [32]. George et al. reviewed 11 cases of COVID19 and IE coinfection; the most common pathogens were Staphylococci and Enterococci; more than half of patients presented with systemic emboli and pulmonary edema [33].

During lockdown periods, emergency department visits decreased by 22.1%, and cardiology admissions dropped by 25.5%, reflecting the profound effects of the COVID-19 pandemic on cardiovascular healthcare access. Only a small percentage of patients evaluated for cardiac conditions tested positive for SARS-CoV-2, with presumed mechanisms of viral cardiac injury including systemic inflammation and cytokine storm, and endothelial damage resulting from both direct viral action and the patient’s systemic status [34]. Due to fear of infection and concerns about healthcare system overload, patients experienced significant delays in seeking care, contributing to increased out-of-hospital cardiovascular mortality [35]. Retrospective studies demonstrated that although in-hospital mortality remained relatively stable, patients admitted during the pandemic presented with more severe disease forms, requiring prolonged hospitalization and more complex surgical interventions [36]. Limited access to timely care, hospital reorganization for COVID-19 patients, and the risk of misdiagnosis or delayed diagnosis contributed to suboptimal therapeutic pathways. An additional source of error arose from the clinical picture dominated by respiratory symptoms (fever, cough, dyspnea, fatigue) typical of viral infections, which delayed the diagnosis of IE and reduced the use of TEE, performed in only 57% of cases [31]. Taghizadeh-Waghefi, in a 4-article case series and systematic review, emphasized the need to diagnose and treat IE in COVID-19 patients as early as possible [37].

A nationwide study including all public and private hospitals in France from January to September 2020 recorded 8128 cases of IE. During the national lockdown (17 March–10 May), hospitalizations slightly decreased (−3.2%), followed by a significant post-lockdown increase (+7.0%). Although viral infection can facilitate IE development, only 3% of endocarditis patients were infected with SARS-CoV-2, suggesting that most cases were not directly related to viral infection. However, in coinfected patients, mortality was twice as high compared to those without COVID-19, indicating a significant clinical impact in associated cases [38].

A relevant case is a 57-year-old male patient with a history of stroke due to a patent foramen ovale closed percutaneously, who was admitted with severe SARS-CoV-2 pneumonia that progressed to septic acute respiratory distress syndrome (ARDS) and confirmed MRSA sepsis. IE was diagnosed by TEE (performed under strict isolation conditions) which showed an aortic valve vegetation and valvular perforation, in the context of high clinical suspicion (persistent fever, positive blood cultures, elevated inflammatory markers). The particular challenge was performing TEE in the contaminated intensive care unit area, using full personal protective equipment [39].

In immunocompromised patients with a history of transplantation and severe COVID-19, the risk of IE caused by uncommon pathogens (e.g., *Corynebacterium striatum*) is increased. When valve replacement surgery is required, rapid deterioration can occur with refractory ARDS necessitating early initiation of veno-venous extracorporeal membrane oxygenation postoperatively [40]. In diabetic patients with a history of recurrent urinary tract infections and recent COVID-19 infection, the risk of developing IE is increased [41].

Compared to the pre-pandemic period, patients in the post-pandemic cohort more frequently presented with mitral valve involvement, comorbidities, and *Staphylococcus aureus* infections, but benefited from a lower rate of postoperative complications such as respiratory failure and low cardiac output syndrome. The time-to-surgery increased after the pandemic began, without a negative impact on mortality [42].

Recent studies indicate that the COVID-19 pandemic had a significant impact on the clinical characteristics, manifestations, and prognosis of hospitalized IE patients. Post-pandemic patients exhibited a higher burden of comorbidities, longer hospital and intensive care unit stays, and significantly increased in-hospital mortality. The incidence of severe complications such as stroke, aspiration pneumonia, and nosocomial myocardial infarction was also higher during the post-pandemic period, suggesting a later and more severe disease presentation. However, patients who underwent valve surgery for IE had stable outcomes and decreased mortality despite the pandemic [43]. COVID-19 was also associated with an increased risk for IE in patients with opioid or cocaine use disorders [44].

**Table 1:** Studies highlighting possible interactions between Infective Endocarditis and COVID-19.

Authors, Year	Title	Study Design and Size	Findings	Limitations
Quintero-Martinez et al., 2022 [31]	*A clinical profile of infective endocarditis in patients with recent COVID-19: A systematic review*	Systematic Review 21 cases	Systemic and endothelial inflammation could lead to IE Avoidance of echocardiography may underdiagnosed IE	Sample Size Retrospective Study
Bele et al., 2023 [32]	*A Comprehensive Review on Cardiovascular Complications of COVID-19: Unraveling the Link to Bacterial Endocarditis*	Review	Convergence of viral-induced endothelial dysfunction, immune dysregulation, and microbial translocation creates a conducive environment for bacterial colonization Early recognition of IE can significantly influence patient outcomes	Narrative Review
Taghizadeh-Waghefi et al., 2023 [37]	*Cardiac Surgery for Treatment of COVID-19-Associated Infectious Endocarditis*	Systematic Review 12 cases	Inflammatory response caused by SARS-CoV-2 may result in damage to the endocardium of native heart valves, a convenient site for bacterial infection Avoid delays in diagnosis and treatment to decrease mortality in patients with IE	Sample Size Retrospective Study
Mikus et al., 2024 [42]	*Surgical Treatment of Active Endocarditis Pre- and Post-COVID-19 Pandemic Onset*	Single Center Cohort Study 535 cases	Incidence of surgically treated IE significantly increases after COVID-19 Higher incidence of mitral valve involvement Mortality and outcomes unaffected	Retrospective Study
Novelli et al., 2023 [43]	*Impact of the COVID-19 Pandemic on Infective Endocarditis Management and Outcomes: Analysis of a National Clinical Database*	National Cohort Study 128,539 cases	Patients with IE after COVID-19 were more complex with worsened outcomes Patients who underwent surgery had stable outcomes and improved mortality despite the pandemic COVID-19 may predispose toward IE	Retrospective Study
Wang et al., 2022 [44]	*Association of COVID-19 with endocarditis in patients with cocaine or opioid use disorders in the US*	National Cohort Study 1,116,125 cases	COVID-19 associated with significantly increased risk for IE in patients with opioid or cocaine use disorders	Retrospective Study

IE: Infective Endocarditis. ARDS: Acute Respiratory Distress Syndrome.

## 6. Diagnosis, Imaging, Current Challenges

Diagnosis of IE is based on a combination of clinical, microbiological, and imaging criteria; the most commonly used are the modified Duke criteria. Given recent technological advances and the accumulation of clinical data over the past years, a comprehensive revision of the Duke criteria for IE diagnosis has become necessary [45]. The primary motivation for these changes is to increase diagnostic sensitivity and specificity, especially in complex cases such as prosthetic-related or culture-negative endocarditis (e.g., NBTE) [46].

Accordingly, the International Society for Cardiovascular Infectious Diseases working group expanded pathological criteria to include modern molecular methods—such as 16S/18S polymerase chain reaction (PCR), fluorescence in situ hybridization (FISH), and metagenomic sequencing—which have proven to be superior to conventional cultures, particularly in cases of prosthetic valve infections or after antibiotic therapy. Additionally, the redefinition of the “typical microorganism” concept was based on the causal association with IE rather than frequency of occurrence. *Enterococcus faecalis* is considered typical regardless of the source, which increases diagnostic sensitivity without reducing specificity [47].

Imaging is an essential aspect in IE diagnosis, with echocardiography as the first-line investigation. TTE is recommended initially, while TEE offers superior sensitivity, especially in cases involving prosthetic valves or suspected complications. In inconclusive or complex cases, multimodal imaging techniques—such as cardiac computed tomography (CCT), Positron Emission Tomography/CT (PET/CT), and magnetic resonance (MRI)—play a complementary role, providing additional information on lesion extent, perivalvular complications, or embolic phenomena [45].

A meta-analysis demonstrated that TEE has significantly higher sensitivity and negative predictive value than CCT for detecting cardiac vegetations, while CCT offers superior specificity in diagnosing prosthetic valve IE and tends to be more sensitive in identifying peri-annular complications [48].

In the absence of standardized criteria, the diagnosis of NBTE requires an integrated approach combining clinical suspicion, serological investigations, and multimodal imaging assessment. TEE remains the imaging reference standard, supplemented by CCT, MRI, and PET/CT for detecting emboli, endocardial inflammation, and differentiating sterile from infectious vegetations. A composite risk index based on clinical, biological, and imaging data has also been proposed for NBTE probability stratification [49].

Due to the increased incidence of prosthetic material IE and the higher risk of complications compared to native valve disease, prompt diagnosis is crucial. Despite limitations imposed by artifacts from prosthetic material, echocardiography remains the first-line diagnostic tool for prosthetic valve endocarditis because of its wide availability, absence of radiation, and rapid assessment capabilities. TEE remains the reference method with superior sensitivity over TTE for detecting vegetations, abscesses, prosthetic dysfunction, premature structural degeneration [50].

An important recent advancement for accurate diagnosis of IE is the addition of a major surgical criterion, which recognizes the value of direct intraoperative observation in the absence of other evidence. Minor criteria have also been updated, including new predisposing conditions such as Trans-catheter Aortic Valve Implantation (TAVI) and CIED), and vascular complications (e.g., cerebral or splenic abscesses) [51].

Given the complexity of IE scenarios, the implementation of a dedicated multidisciplinary team (Endocarditis Team) has been associated with significant clinical benefits, including reduced in-hospital and one-year mortality, increased rates of early surgical interventions, and decreased complications such as stroke, severe heart failure, and cardiac abscess [52]. In recent studies, five-year survival was significantly higher among patients managed through an interdisciplinary approach [53]. This strategy led to a change in therapeutic decisions in 44% of cases by refining the diagnosis, recommending additional investigations, and re-evaluating surgical indications, highlighting the importance of collaboration between specialties and coordination with referral centers to optimize prognosis [52,53].

## 7. Discussion

### 7.1. Rising Incidence and Changing Epidemiology

IE is a complex and heterogeneous pathology. Its scenario is continuously evolving both from a microbiology standpoint (new bacteria, new antibiotic resistance) and a clinical one (younger patients, older patients with risk factors).

Etiology of IE has undergone significant changes over the past two decades. Whereas subacute infections were previously the most common (caused by *Streptococcus viridans*), *Staphylococcus aureus* acute forms—often Methicillin-Resistant (MRSA)—now predominate, especially among PWID and CIED carriers. Concurrently, there is a rise in enterococcal IE cases, particularly following procedures such as TAVI in elderly patients [9,53]. In high-income countries, prosthetic material- and device-related cases are increasingly common, often in healthcare-associated settings. Early infections (≤60 days) are usually caused by nosocomial pathogens, while late-onset cases more often involve streptococci or enterococci. Healthcare-associated IE accounts for ~25–30% of cases, with *Staphylococcus aureus* (~26.6%), viridans group streptococci, other streptococci, and enterococci responsible for up to 90%. Less frequent pathogens include the HACEK (Haemophilus, Aggregatibacter, Cardiobacterium, Eikenella, Kingella) group (1.1–1.4%) and fungi—mainly *Candida* spp.—(1.2–1.5%) [17,54,55].

The increasing incidence of cases (including NBTE) and post-COVID-19 peculiar conditions reflect not only epidemiological changes but also the adaptability of both the pathogen and the host, particularly if the latter has an altered immune system. While rheumatic fever and CHD were historically the primary causes of IE, the recent incidence rise is mainly attributed to at-risk populations.

Age at the time of diagnosis has increased over the years [10]. This shift underscores the epidemiological transition from classical valvular disease forms, predominantly rheumatic, to degenerative etiologies typical of the elderly population. As a result, the current IE population is composed of vulnerable patients often with multiple comorbidities or compromised immune status [11].

Socio-economic context is crucial in understanding the rising incidence and prognostic disparities in IE, suggesting the need for targeted public health interventions and diagnostic methods adapted to regional specificities. Care for patients with IDU IE entails substantial costs, with a high rehospitalization rate (~50%) and fewer than 6% receiving addiction treatment post-discharge [56]. These patients often face lack of addiction management during hospitalization, difficulties in multidisciplinary coordination, stigma from healthcare personnel, and post-hospitalization absenteeism. An innovative model integrating addiction treatment into clinical management for IDU-IE patients is the Multidisciplinary Endocarditis Evaluation Team (MEET), developed at Yale School of Medicine. MEET comprises multiple specialists collaborating regularly to ensure a unified treatment plan addressing both infection and substance use disorder, demonstrating reduced overall mortality, lower readmission rates, decreased overdose risk, and improved post-discharge survival [57].

### 7.2. Personalized Antibiotic Prophylaxis

Studies about antibiotic prophylaxis support the current approach favoring limited prophylaxis and emphasizing the need for rigorous oral hygiene. Current European guidelines stratify population in high-risk, intermediate-risk, and low-risk patients; high-risk patients must receive prophylaxis before dental procedures; it may be considered for invasive diagnostic or therapeutic procedures [58]. However, the etiological shift suggests that antibiotic armamentarium should be reconsidered; individualized risk stratification should be implemented when performing invasive procedures. In the next future, IE prophylaxis should be further stratified for high-risk population, choosing a specific prophylaxis strategy based on patient, procedure, antibiotic resistance and tolerance in each region. For instance, a young patient with CHD undergoing bronchoscopy in North America could benefit from a different prophylaxis strategy compared to an elderly patient with CIED in Europe who undergoes dental extraction. This “personalized” strategy would also decrease antibiotic resistance and tolerance. Nevertheless, the only way to ultimately achieve this goal is to design specific regional/national epidemiologic studies, having as a result complete and accurate information regarding each specific at-risk population.

Data about CHD suggest that both the severity of the cardiac malformation and the associated therapeutic interventions significantly contribute to the vulnerability of CHD patients to IE, highlighting the need for effective screening and management [18,19]. Additionally, risk stratification based on the type of congenital heart lesion and cardiovascular intervention history could be helpful.

### 7.3. NBTE: Rule out in High-Risk Population

NBTE is insidious and clinically deceptive. It may present nonspecific clinical manifestations and be easily masked by other initial diagnoses, delaying identification of the primary pathological mechanism [20,21,22,23]. Given the subtle nature, integrating each patient’s specific clinical features is essential (particularly in patients with a prothrombotic status and autoimmune diseases). If endocarditis is suspected and blood cultures and PCR are negative, but predisposing conditions are present (autoimmune disease, prothrombotic status, tumors), the patient should be considered high-risk for NBTE. Correlating this individualized clinical profile with multimodal imaging findings can significantly enhance diagnostic accuracy. TEE should be performed every time TTE is non-diagnostic in a high-suspicion scenario [26,59]. Cardiac and whole-body CT are crucial to detect local complications (coronary embolism) and systemic complications, respectively [59,60]. Moreover, whole-body CT scan may identify a primary or metastatic tumor. NBTE has been diagnosed after COVID-19 as well, as COVID-19 infection may play a role leading to a hypercoagulation status [28,29]. After NBTE diagnosis, patients should receive anti-coagulant therapy to decrease the risk of recurrence and systemic emboli; most common strategy is lifelong warfarin therapy [28]. NBTE remains a rare entity, but post-mortem diagnosis suggests its underdiagnosed nature. We should focus on high-risk patients (performing a multimodal diagnostic process) to rule out or get a prompt diagnosis, and post-diagnosis anticoagulant therapy to decrease complications.

### 7.4. COVID-19 and IE: Never Postpone Diagnostic Process

COVID-19 pandemic had an indirect (as no direct cause-effect link has been described so far) but significant effect on IE diagnosis and management, leading to delayed diagnosis and treatment. Available studies emphasize the need for diagnostic and care systems capable of effective functioning even during health crises, and able to differentiate two different pathologies in the context of infectious syndromes. Reviews highlighted the need for early diagnosis to avoid poor outcomes [32,33,37]. Also, significant changes in the clinical and microbiological profile of IE after the onset of the pandemic have been described [40,42]. TEE and multidisciplinary team have proven crucial, even in extraordinary social and health crises, to achieve good results [31,37,39]. Some authors hypothesized that COVID-19 could increase the risk of IE [32]. However, the pandemic has ended recently and very few data are available regarding a specific correlation between these two infections. Accurate and long-term studies will be crucial in the future to better understand and determine if the SARS-CoV-2 effectively played a direct role in facilitating IE and its potential long-term effects in IE patients. Also, it is not yet known whether COVID-19 could be a risk factor for IE (or other cardiovascular diseases) in the long term. Future studies should focus on the possible pathophysiologic link between the two infections and investigate potential long-term complications. Moreover, it will be necessary to assess IE incidence in the coming years: will it increase? Among IE patients, what proportion will have had previous COVID-19 infection? Will certain IE etiologies become more frequent? Will prior COVID-19 infection lead to more severe IE? Although the COVID-19 pandemic is over, it is too early to answer these questions.

## 8. Limitations

This study has limitations. This is a narrative review which includes studies published after 2014, searched through a single database. A narrative review has lower reproducibility and is less comprehensive and exhaustive than a systematic review. Our review also included case reports, whose statistical power is obviously lower than cohort studies and systematic reviews. Some studies refer to national or continental data, limiting their external validity. Future, more robust studies on NBTE are needed to better understand its pathological mechanisms and improve diagnosis and clinical management. Studies on long-term COVID-19 effects on IE and cardiovascular system are still ongoing and not yet fully available.

## 9. Conclusions

The current evolving IE scenario justifies the ongoing need to update diagnostic criteria and clinical practice guidelines, establish an Endocarditis multidisciplinary team, and integrate advanced imaging techniques. These factors can contribute to improving diagnostic accuracy and clinical outcomes in an increasingly nuanced clinical context. NBTE should be ruled out in at-risk patients. COVID-19 and IE may be linked, but more accurate and long-term data are required.

## Data Availability

No new data were created or analyzed in this study. Data sharing is not applicable.

## Abbreviations

The following abbreviations are used in this manuscript:

APLAS	Antiphospholipid Antibody Syndrome
ARDS	Acute Respiratory Distress Syndrome
BD	Behçet’s disease
CCT	Cardiac Computed Tomography
CHD	Congenital Heart Disease
CIED	Cardiac Implantable Electronic Device
FISH	Fluorescence In Situ Hybridization
HACEK	Haemophilus, Aggregatibacter, Cardiobacterium, Eikenella, Kingella
HCV	Hepatitis C Virus
IE	Infective Endocarditis
IDU	Intravenous Drug Use
MRI	Magnetic Resonance
MEET	Multidisciplinary Endocarditis Evaluation Team
MRSA	Methicillin-Resistant Staphylococcus Aureus
NBTE	Non-Bacterial Thrombotic Endocarditis
PET/CT	Positron Emission Tomography/Computed Tomography
PWID	People Who Inject Drugs
PCR	Polymerase Chain Reaction
SDI	Socio-Demographic Index
SLE	Systemic Lupus Erythematosus
TEE	Trans-Esophageal Echocardiogram
TTE	Trans-Thoracic Echocardiography
TAVI	Transcatheter Aortic Valve Implantation
